# A reproducible EEG hyperscanning dataset for triadic social decision-making during an iterated 3-player Prisoner’s Dilemma

**DOI:** 10.1016/j.dib.2026.113064

**Published:** 2026-07-10

**Authors:** H. Kim, S.C. Jun, C.S. Nam

**Affiliations:** a Department of AI Convergence, Gwangju Institute of Science and Technology, Gwangju, 61005, South Korea; b Center of Excellence in Product Design and Advanced Manufacturing, North Carolina A&T State University, Greensboro, NC, 27411, USA

**Keywords:** EEG hyperscanning, Triadic interaction, Prisoner’s Dilemma, Inter-brain synchrony, Phase-locking value, Event-related potentials

## Abstract

This data article describes an open EEG hyperscanning dataset acquired during an iterated 3-player Prisoner’s Dilemma (PD) task, designed to support reproducible research on triadic social decision-making. EEG was recorded simultaneously from three participants per group (11 groups; 33 subjects) using synchronized acquisition, with decision-locked and feedback-locked epochs provided for 40 task trials per subject and three 60-s resting-state runs per group. The released BIDS format contain 19-channel EEG arrays sampled at 300 Hz, with explicit epoch definitions for decision (−1000 to 4000 ms) and feedback (−1000 to 2000 ms) periods, as well as trial-wise behavioral choice labels (1=cooperate, 2=defect) enabling reconstruction of dyad- and triad-level outcomes. Questionnaire metadata (personal information and pre/mid/post state measures) Yare also provided as supplementary spreadsheets. To facilitate reuse and benchmarking, we release Python code that reproduces the preprocessing pipeline (average reference, FIR filtering, ICA + ICLabel for task EEG), ERP summaries, and inter-brain synchrony measures (PLV and coherence) with window-matched resting baselines and cluster-permutation statistics. The dataset is intended for method development and benchmarking in ERP analysis, inter-brain synchrony estimation, and modeling of dynamic group interaction states in triadic games.

Specifications Table

**Table:** 

Subject	Biology
Specific subject area	Neuroscience; Social neuroscience; Cognitive neuroscience
Type of data	Raw and epoched EEG data; behavioral choice matrix; questionnaire responses; metadata; analysis code. Data formats: BIDS (.set), Excel (.xlsx), CSV, Python scripts, figures, and summary tables.
Data collection	EEG hyperscanning data were collected from 11 triads (33 participants) during an iterated 3-player Prisoner’s Dilemma task. EEG was recorded using three synchronized DSI-24 systems (Wearable Sensing; 19 channels per participant; 300 Hz) via OpenViBE. Behavioral choices were recorded trial-by-trial, and questionnaires were administered before, during, and after the task.
Data source location	Gwangju Institute of Science and Technology (GIST), Gwangju, Republic of Korea.
Data accessibility	Repository name: OpenNeuro Data identification number: 10.18112/openneuro.ds007822.v1.0.0 Direct URL to data:https://doi.org/10.18112/openneuro.ds007822.v1.0.0 Instructions for accessing these data: The dataset is publicly available through the OpenNeuro DOI. The repository includes BIDS files, behavioral annotations, questionnaire data, metadata, and supporting files
Related research article	None.

## 1 Value of the Data

• Provides an open, event-synchronized EEG hyperscanning dataset for triadic (3-person) interaction, enabling analyses beyond dyadic decompositions.
• Supports reproducible benchmarking of ERP and inter-brain synchrony (IBS) methods through shared data structures, preprocessing code, and statistical evaluation scripts.
• Includes trial-wise behavioral labels aligned to EEG epochs, allowing direct mapping between neural activity and dyad/triad decision outcomes.
• Includes pre/mid/post questionnaire metadata that can be used as covariates for inter-subject variability, fatigue/stress, and data quality sensitivity analyses.
• Can be used to develop and compare computational models of dynamic cooperation states and belief updating in repeated social dilemmas.

## 2 Background

Social decision-making often requires individuals to adapt their choices according to the actions and feedback of others during repeated interaction. The iterated Prisoner’s Dilemma has been widely used as a controlled framework for studying cooperation, defection, reciprocity, and history-dependent decision strategies [1]. In neuroscience, hyperscanning enables simultaneous recording of neural activity from multiple interacting individuals and has been applied to investigate interpersonal neural coupling during social interaction [2,3]. EEG hyperscanning is particularly useful for studying dynamic interaction because it provides millisecond-level temporal resolution and supports event-locked and frequency-specific analyses.

This dataset was compiled to provide an open EEG hyperscanning resource for triadic social decision-making. Three participants simultaneously performed an iterated 3-player Prisoner’s Dilemma while EEG was recorded using synchronized devices. The dataset includes decision-locked and feedback-locked EEG epochs, resting-state EEG, trial-wise behavioral choices, questionnaire responses, metadata, and analysis code. It was organized to support reuse in event-related potential analysis, inter-brain synchrony estimation, behavioral modeling, and methodological benchmarking for small-group social neuroscience.

## 3 Data Description

Data are released as one BIDS format. Each file contains a struct with task EEG epochs, resting EEG, channel metadata, epoch limits, preprocessing metadata, and trial-wise behavioral choices. The dataset is available on OpenNeuro (DOI: https://doi.org/10.18112/openneuro.ds007822.v1.0.0).

### 3.1 File organization and naming

**Fig. 1:**
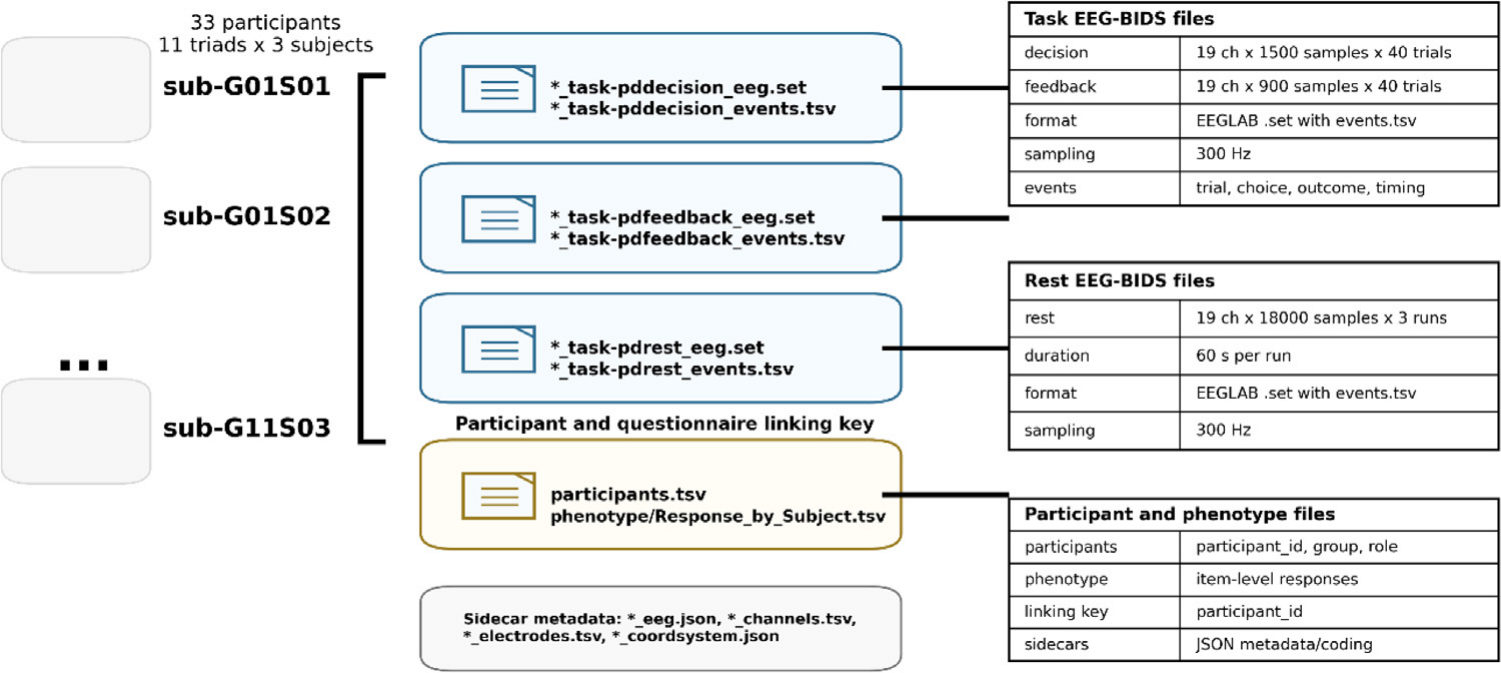
EEG-BIDS directory structure and relationships among EEG, behavioral, and phenotype files.

G{01-11}S{01-03}_eeg.set(BIDS struct `data`) (Fig. 1)

### 3.2 Variables in data file

**Table:** 

Field	Type	Shape	Description
decision_X	single	19×1500×40	Decision-locked epochs (−1000 to 4000 ms; 300 Hz)
feedback_X	single	19×900×40	Feedback-locked epochs (−1000 to 2000 ms; 300 Hz)
resting	double	19×18000×3	Resting EEG (3 runs; 60 s each)
srate	numeric	1×1	Sampling rate (300 Hz)
ch_locs	cell	1×19	Channel labels/locations for 19-channel montage (per subject block)
decision_ep	numeric	1×2	Epoch limits in ms for decision_X
feedback_ep	numeric	1×2	Epoch limits in ms for feedback_X
preproc	struct	1×1	Preprocessing metadata (pipeline parameters)
score	double	40×3	Trial-wise choices (1=C, 2=D) for S1–S3
score_str	cell	40×3	String-form choices aligned with score (e.g., `C'/`D')

## 4 Experimental Design, Materials and Methods

### 4.1 Method

The dataset was generated from a triadic EEG hyperscanning experiment in which three participants simultaneously performed an iterated 3-player Prisoner’s Dilemma task. The following sections describe the participants, task structure, EEG acquisition, preprocessing pipeline, behavioral annotations, and validation analyses.

### 4.2 Participants

EEG data were collected during a sequential Prisoner’s Dilemma (PD) game involving 11 groups (11 groups × 3 players), comprising a total of 33 participants (13 females; mean age = 22.59 ± 2.56 years). All participants were right-handed, had normal or corrected-to-normal vision, and reported no history of neurological or psychiatric disorders. All participants were naïve to the Prisoner’s Dilemma task and had no prior experience with similar experimental paradigms. The study protocol was approved by the Institutional Review Board (IRB) of the Gwangju Institute of Science and Technology (GIST) (approval no. 20231207-HR-74-02-02), and all participants provided written informed consent prior to participation.

### 4.3 EEG acquisition and hyperscanning setup

EEG recordings were obtained using three DSI-24 devices from Wearable Sensing, which each feature 24 dry electrodes (including the standard 19 channels, two earlobes, and three additional channels). These devices are configured based on the standard 10/20 system (Fig. 3) and utilize a sampling rate of 300 Hz. Reference electrodes were positioned on both earlobes (A1, A2). Prior to each recording session, electrode impedance and signal quality were verified using the device interface, and all channels were confirmed to be within acceptable operating ranges.

The synchronization of EEG data collection with task events from the three devices was accomplished using OpenViBE [4]. The three DSI-24 EEG streams were acquired independently using separate OpenViBE acquisition processes. Task-event markers were transmitted from the experimental program to each acquisition process through TCP/IP and were inserted as software markers into the corresponding EEG data streams. The three acquisition processes therefore received a common event message, but they did not share a hardware clock or a physical trigger line.

OpenViBE was configured with a software-latency tolerance of 2 ms. The acquisition procedure was set to terminate automatically if the monitored latency exceeded this threshold, and no latency-related termination occurred during data collection. The 2-ms value therefore represents the predefined software tolerance used during acquisition rather than an empirically measured mean or maximum inter-device timing offset. No independent hardware-based timing validation, such as a simultaneous TTL pulse, loopback measurement, or oscilloscope assessment, was performed. Accordingly, exact mean and maximum synchronization errors across the three systems are not available.

This synchronization framework ensures precise temporal alignment of neural signals across participants, which is critical for reliable estimation of inter-brain synchrony (IBS). The recorded EEG data underwent a high-pass filter at 1 Hz using a 4th order Butterworth filter, and a 60 Hz notch filter was applied to eliminate power line noise. All filtering steps were applied consistently across subjects to ensure comparability in downstream analyses.

### 4.4 Experimental design

**Fig. 2:**
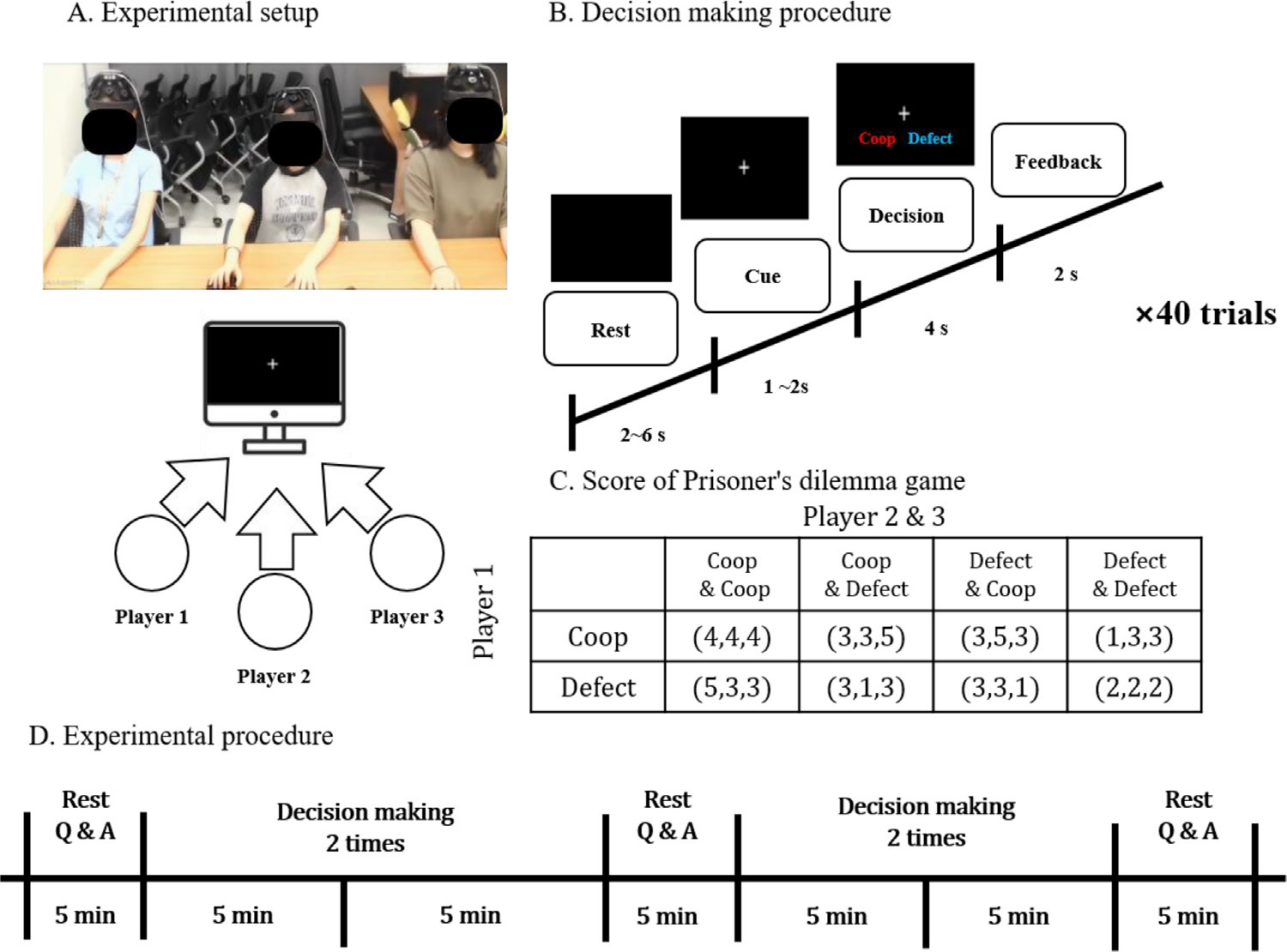
Overview of the experimental workflow. (A) The 3-person prisoner's dilemma game was set up for experimentation. Simultaneous EEG data acquisition was conducted using three synchronized EEG-recording devices facilitated by OpenViBE. (B) The single trial procedure. (C) The payoff matrix for the iterated Prisoner's Dilemma Game. (D) The experimental procedure.

In this experiment, an iterated 3-player prisoner's dilemma (PD) game was implemented [5]. The payoff for each interaction was weakly dominated, aiming to encourage cooperative decision-making throughout the iterations [6] (see Fig. 2, C). Participants were compensated based on their performance across 40 rounds, with a maximum reward of 30,000 Korean won (approximately $22) for those who scored 190 points or more. The reward decreased by 3,000 won for each 15-point reduction in their score, creating a tiered payment system based on performance. This incentive structure was designed to maintain engagement and promote meaningful strategic decision-making across trials. Points were accumulated across all 40 trials, and performance-dependent compensation was determined from each participant’s final cumulative score. Participants who obtained 190 points or more received the maximum compensation of 30,000 KRW. Compensation was reduced by 3,000 KRW for each 15-point decrease below this threshold.

**Fig. 3:**
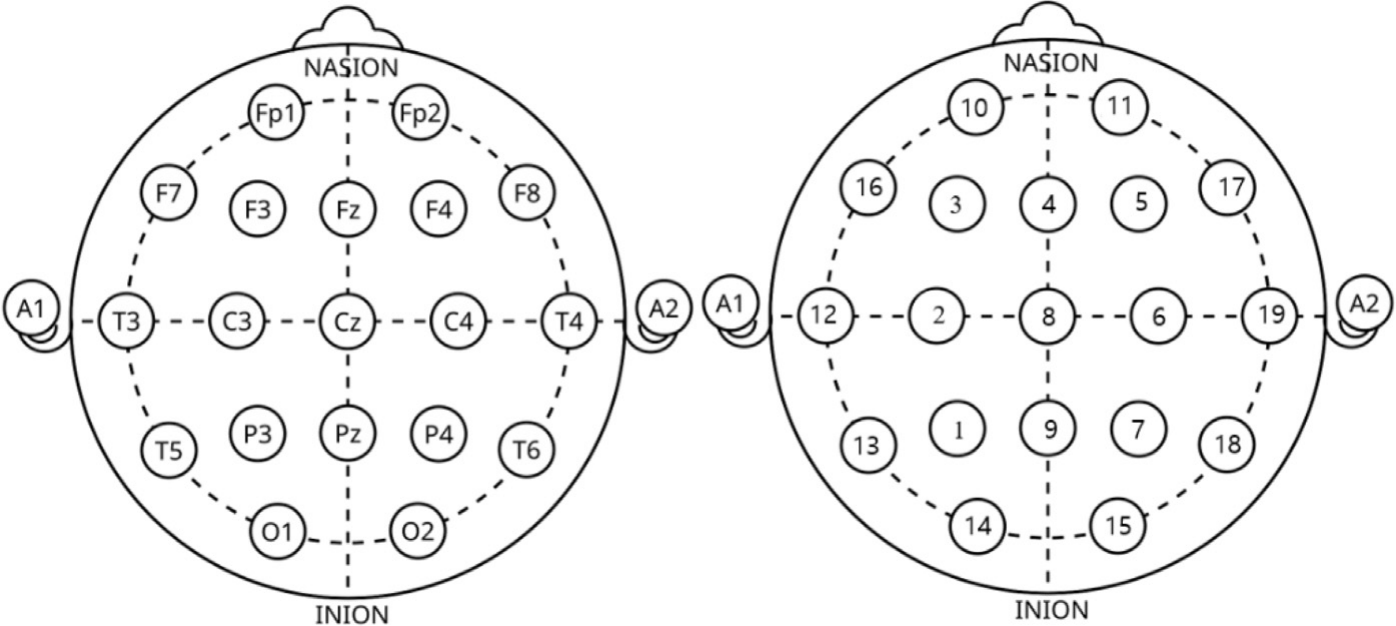
The channel configuration of the International 10-20 system (19 EEG and both earlobes recording electrodes). The right panel corresponding location of each electrode, and the left panel indicates the indexing.

To study social group synchrony during the decision-making process, each group of 3 players participated in four rounds of the PD game, comprising ten consecutive trials. The experiment (Fig. 2) was structured as follows: a 1-2 second instruction phase, a 4-second decision-making phase, a 2-second feedback phase presenting the decision outcome and scores, followed by a 2-6 second resting phase ([cue(2s) + decision(4s) + feedback(2s) + rest(2∼6s)] x 10 trials x 4 repetitions). This temporally structured design enables precise event-locking of neural responses, facilitating both ERP analysis and time-resolved inter-brain synchrony estimation. The scoring and reward system of the PD game is depicted in Fig. 2B. During each trial, the three participants made their choices independently within the same 4-s decision window. Participants used separate keyboards that were visually shielded from the other players, and keypress sounds were minimized so that an individual choice could not readily be inferred before the feedback phase. Participants were instructed not to engage in verbal or non-verbal communication after the experiment began.

After all three responses had been submitted, a common feedback screen was presented simultaneously to the three participants. The screen displayed the completed trial outcome, the choices made by all three participants, and each participant’s cumulative score up to that trial. Thus, participants had access to the other players’ previous choices and cumulative performance during the feedback phase, but not to their ongoing responses during the decision phase.

Additionally, resting-state EEG was recorded for 60 seconds before and after the experiment. Resting-state recordings were included to provide baseline estimates for inter-brain synchrony and spectral analyses. These baseline recordings enable normalization of task-related synchrony measures and control for non-task-related coupling effects.

Personal and baseline questionnaire data were collected before the task. These measures included demographic and health-screening information, prior experience with game theory and related experimental paradigms, prior familiarity among group members, recent sleep and behavioral-state covariates, baseline subjective state, social cynicism, and generalized interpersonal trust.

A mid-experiment questionnaire was administered between task blocks to assess subjective state, visual and physical discomfort, mental fatigue, interpersonal perception and trust, interaction quality, task concentration, and satisfaction with the ongoing results. Corresponding state-dependent items were administered again after completion of the task. All questionnaire responses were linked to the EEG and behavioral data using the BIDS participant_id.

### 4.5 Data availability

The dataset is publicly available on OpenNeuro (DOI: https://doi.org/10.18112/openneuro.ds007822.v1.0.0). The repository contains BIDS format, questionnaire spreadsheets, behavioral annotations, and metadata required for reuse.

### 4.6 Code availability

The analysis code is available at: https://github.com/heegyukim4043/PD_EEG_hyperscan_processing.

The repository provides a fully reproducible workflow to generate the main technical validation outputs from the released BIDS, including (i) preprocessing to create a cleaned cache (cleaned_eeg.pkl), (ii) inter-brain synchrony (IBS) analyses using PLV and circular correlation coefficient (ccorr) with cluster-based permutation testing, and (iii) ERP analyses for decision- and feedback-locked epochs. All scripts are organized to enable direct execution with minimal configuration, supporting full reproducibility of the reported results. The repository is structured to allow users to reproduce, verify, and extend all analyses from raw data to final outputs.

***Preprocessing (bids.py):*** loads BIDS (.set) and produces results/cleaned_eeg.pkl after average referencing, band-pass filtering, and ICA-based artifact rejection for task EEG (Infomax ICA + ICLabel; eye blink/muscle, probability > 0.80). Resting EEG is processed with filtering and average reference (no ICA). This step generates a standardized intermediate dataset that serves as the input for all subsequent analyses and ensures consistency across the pipeline.

***IBS analysis (ibs_analysis.py):*** computes dyad-wise IBS for all within-triad pairs (S1–S2, S1–S3, S2–S3) in the decision and feedback epochs using 0–1000 ms and 0–2000 ms windows, with a window-matched resting baseline and cluster-permutation tests (n=2000, two-tailed). Outputs include figures and per-band summary CSV tables. This analysis design enables statistically robust and comparable estimation of inter-brain synchrony across conditions.

### 4.7 Technical validation

#### 4.7.1 Preprocessing

EEG preprocessing was conducted using the MNE library [7]. The preprocessing pipeline was structured to ensure consistency across subjects and reproducibility of all downstream analyses. Overview of the EEG preprocessing branches for task and resting data. Raw EEG (11 groups × 3 subjects × 19 channels; 300 Hz) underwent a common preprocessing stage (1 Hz high-pass FIR, 60 Hz notch, mastoid reference) saved in “set” files. Task EEG was re-referenced to average reference, filtered (1–100 Hz), and denoised using Infomax ICA with ICLabel-based rejection of eye-blink and muscle components (p > 0.80) [8]. ICA component rejection was performed using a standardized probabilistic threshold to minimize subjective intervention and ensure reproducibility.

Resting EEG used average reference and 1–45 Hz filtering without ICA. This design choice reflects a trade-off between computational efficiency and preservation of continuous resting-state dynamics. Cleaned data were used for ERP extraction and IBS computation; resting IBS baselines were computed using window-matched non-overlapping segments. All preprocessing steps were applied identically across subjects to enable valid cross-subject and cross-group comparisons.

#### 4.7.2 EEG signal quality validation

EEG data quality was evaluated at the participant, task, and channel levels. Trial and run retention was assessed by comparing the number of EEG epochs with the corresponding event annotations. All 33 participants retained the complete set of 40 decision-locked epochs, 40 feedback-locked epochs, and three 60-s resting-state runs. No trial-level epochs were removed from the released dataset; artifact handling for task EEG was performed at the channel and independent-component levels.

**Fig. 4:**
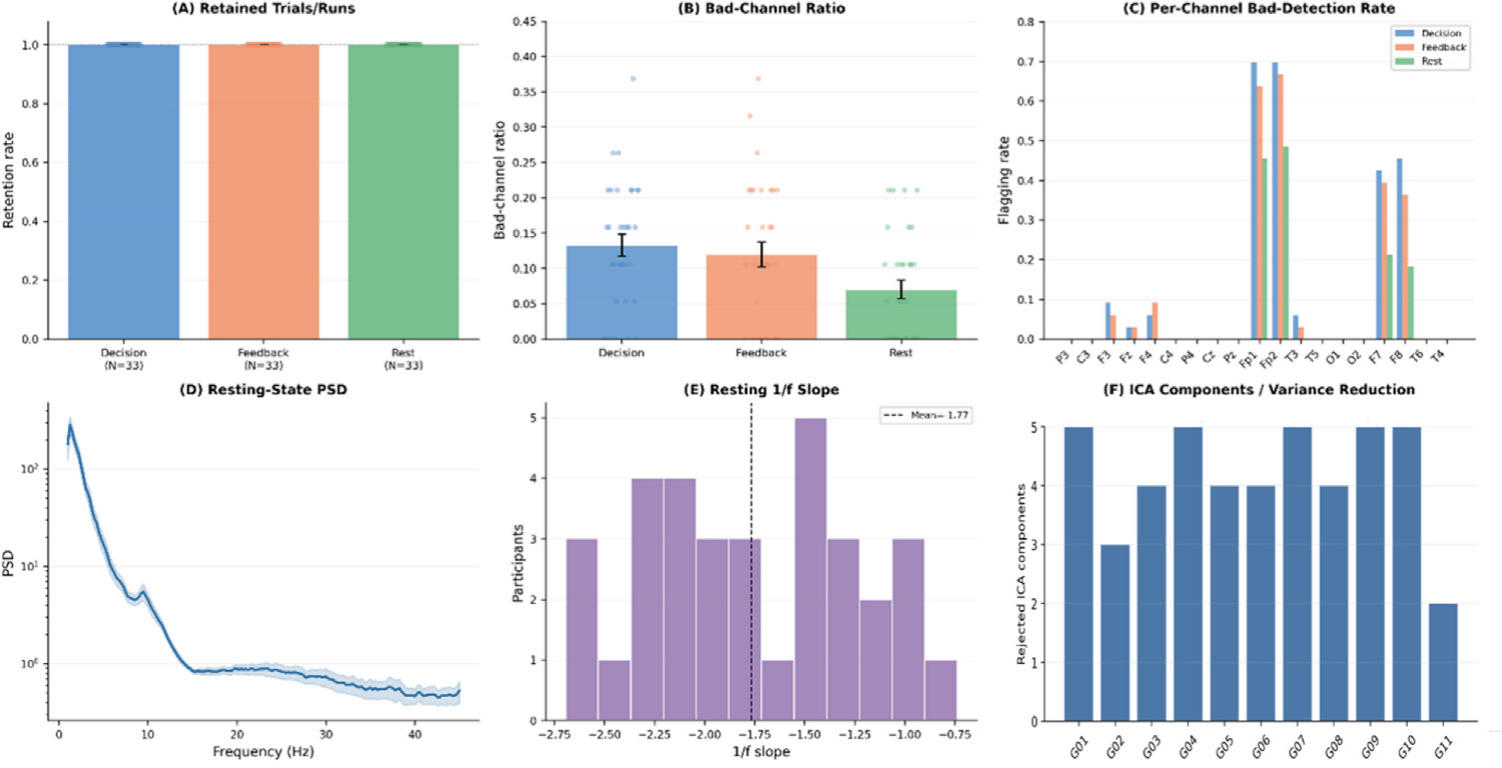
EEG signal-quality validation across participants and recording types. (A) Retention of decision epochs, feedback epochs, and resting-state runs. All participants retained 40 decision epochs, 40 feedback epochs, and three resting-state runs. (B) Distribution of participant-level bad-channel ratios for decision, feedback, and resting-state recordings. (C) Proportion of recordings in which each EEG channel was flagged by the predefined channel-quality criterion. Frontal channels, particularly Fp1, Fp2, F7, and F8, showed the highest flagging rates. (D) Grand-average resting-state power spectral density from 1 to 45 Hz; the solid line represents the participant mean and the shaded region represents. (E) Distribution of participant-level slopes of the resting-state aperiodic spectrum in log coordinates. (F) Number of rejected ICA components summary for task EEG.

Channel quality was evaluated separately for decision, feedback, and resting-state recording (Fig. 4). Across participants, the mean number of channels flagged per 19-channel recording was 2.52 ± 1.73 for decision EEG, 2.27 ± 1.94 for feedback EEG, and 1.33 ± 1.43 for resting-state EEG.

Channel-level flagging was concentrated primarily in the frontal electrodes. Fp2 and Fp1 were flagged in 61.6% and 59.6% of recordings, respectively, followed by F7 and F8 in 34.3% and 33.3% of recordings. This distribution is consistent with the greater sensitivity of frontal dry electrodes to general ocular activity, facial muscle activity, and electrode-contact variability. These channels were not uniformly excluded across the dataset; quality status was determined separately for each participant and recording.

Resting-state spectral quality was evaluated using the power spectral density from 1 to 45 Hz and the slope of the aperiodic spectral component. The grand-average spectrum showed the expected frequency-dependent decrease in power together with a local alpha-range peak. The mean resting-state log–log spectral slope was −1.77 ± 0.55 across participants, with values ranging from −2.69 to −0.74. Participant-level, task-level, and channel-level QC summaries are provided with the analysis outputs to support transparent assessment and user-defined exclusion criteria.

#### 4.7.3 Inter brain synchrony

**Fig. 5:**
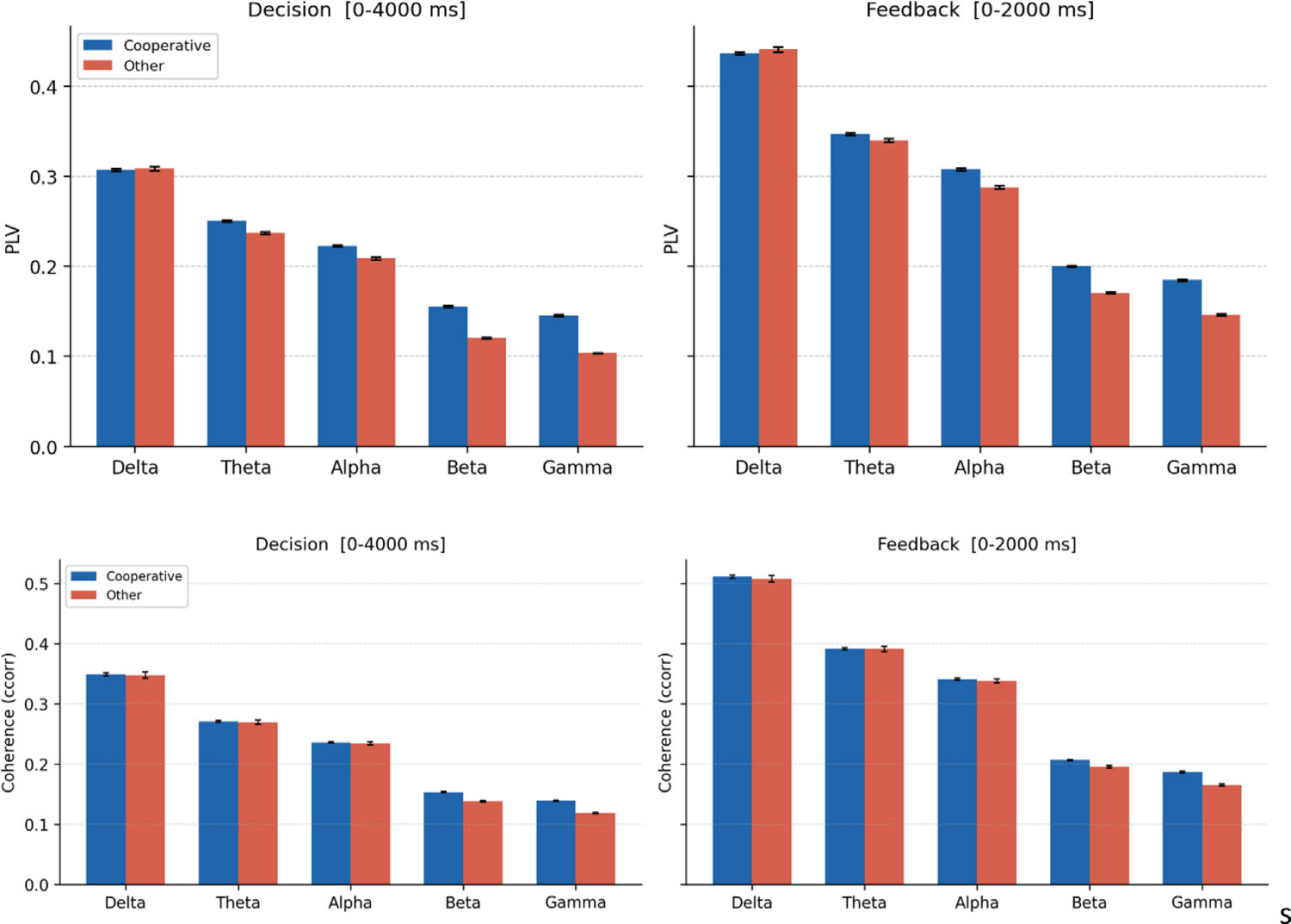
Inter-brain synchrony estimated using phase-locking value and circular correlation. Inter-brain synchrony was quantified for Cooperative trials, defined as trials in which all three participants selected cooperation (CCC), and Other trials, comprising all remaining triadic outcomes. Phase-locking value (PLV) is shown for the decision period from 0 to 4,000 ms (A) and the feedback period from 0 to 2,000 ms (B). The circular correlation coefficient is shown for the corresponding decision (C) and feedback (D) periods. Values were calculated within the delta (1–3 Hz), theta (4–7 Hz), alpha (8–12 Hz), beta (14–25 Hz), and gamma (30–45 Hz) bands.

Inter-brain synchrony was evaluated using phase-locking value (PLV) as the primary illustrative metric and the circular correlation coefficient as a complementary phase-based connectivity measure [9] (Fig. 5). PLV quantifies the consistency of phase differences across observations, whereas circular correlation estimates the association between the instantaneous phase angles of two signals. The two measures therefore characterize related but non-identical aspects of inter-participant phase coupling.

Both metrics were computed using the same preprocessing procedures, behavioral conditions, frequency bands, decision and feedback windows, and within-triad participant pairs. These analyses are provided as technical validation examples demonstrating that the released dataset supports reproducible estimation using multiple inter-brain synchrony metrics. Because both PLV and circular correlation are phase-based measures and may be influenced by common sensory input, shared event timing, and zero-lag components, the results should not be interpreted as definitive evidence of direct or causal neural coupling. The released dataset can also support alternative connectivity measures, including coherence, imaginary coherence, and weighted phase-lag index.

IBS was computed for all within-triad dyads (S1–S2, S1–S3, S2–S3) during both decision and feedback epochs. To enable triadic-level analysis, pairwise IBS measures were retained and can be aggregated to characterize group-level interaction structure. This approach allows investigation of higher-order interaction dynamics by examining how pairwise coupling patterns jointly evolve within the triad. IBS was evaluated within task-relevant time windows aligned to event onset and compared against resting-state baselines constructed using window-matched non-overlapping segments. The use of window-matched resting baselines enables normalization of task-related synchrony and controls for non-task-related coupling effects.

Statistical significance of IBS effects was assessed using cluster-based permutation testing, enabling control for multiple comparisons across channels and frequency bands. Cluster-based permutation testing provides a robust, non-parametric framework for identifying spatiotemporally structured IBS effects while controlling family-wise error rates. These analysis choices are particularly suited for hyperscanning datasets with high temporal resolution, enabling consistent and reproducible quantification of inter-brain coupling across participants and experimental conditions.

#### 4.7.4 Behavioral validation: computation of behavioral indices

**Fig. 6:**
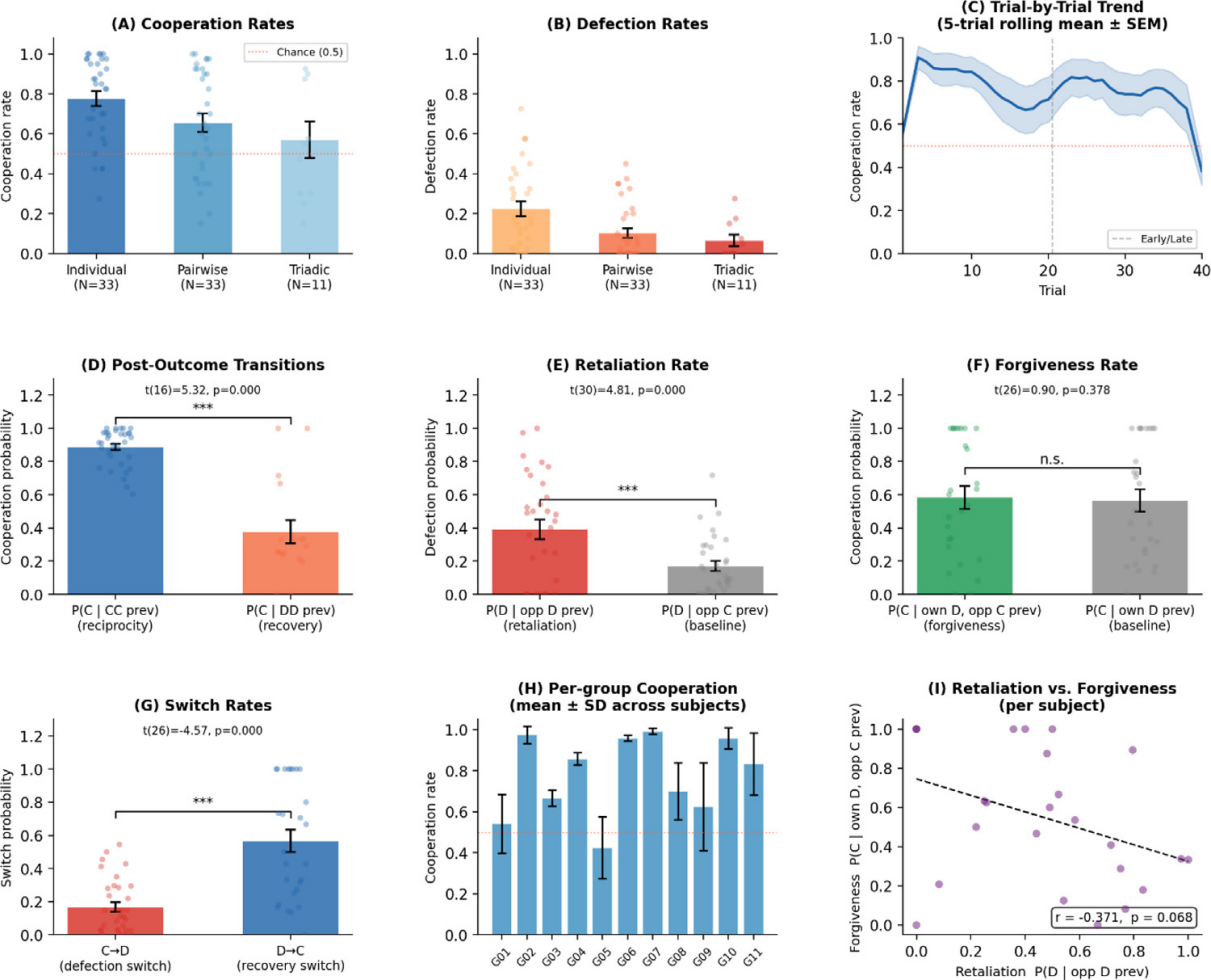
Behavioral results in the iterated Prisoner’s Dilemma (N = 11 groups, 33 subjects, 40 trials). (A) Cooperation rates at three aggregation levels (individual, pairwise, triadic); dashed line indicates chance level (0.5). (B) Defection rates at the same levels. (C) Trial-by-trial cooperation trend (5-trial rolling mean ± SEM) with early/late split at trial 20. (D) Post-outcome transition probabilities reflecting reciprocity (P(C | CC prev)) and recovery (P(C | DD prev)). (E) Retaliation rate (P(D | opp D prev)) compared with baseline (P(D | opp C prev)). (F) Forgiveness rate (P(C | own D, opp C prev)) compared with baseline (P(C | own D prev)). (G) Switch probabilities for C→D and D→C. (H) Per-group cooperation rates (mean ± SD across subjects). (I) Relationship between retaliation and forgiveness across subjects with Pearson correlation.

Behavioral choices were obtained from the trial-wise decision labels stored (40 trials × 3 subjects per group), where each entry indicates the subject’s choice at each trial. Unless otherwise specified, indices were computed per subject and then summarized across subjects; all descriptive and inferential statistics are reported in Table 1, while Fig. 6 provides the corresponding visualizations.

**Table 1:** Summary of effective sample sizes

Figure 6 panel	Comparison	Full N	Valid N	df	t	p
D	P(C | CC prev) vs P(C | DD prev)	33	17	16	5.32	< .001
E	P(D | opp D prev) vs P(D | opp C prev)	33	31	30	4.81	< .001
F	P(C | own D, opp C prev) vs P(C | own D prev)	33	27	26	0.90	.378
G	C→D switch rate vs D→C switch rate	33	27	26	−4.57	< .001

**Cooperation/defection rates at multiple social scales**: Behavioral indices were computed from the trial-wise choice matrix score (40 trials × 3 subjects per group), where each entry indicates a subject’s choice at a given trial [10]. Choices were coded as C (cooperate) or D (defect). All indices were computed from consecutive trials (t−1 → t) where applicable. Unless otherwise specified, behavioral indices were first computed per unit (subject, dyad, or group) and then summarized across the corresponding units for visualization in Fig. 4; inferential statistics are reported in Table 1.

**Individual cooperation and defection rates**: Individual cooperation rate was defined for each subject as the proportion of trials [11] in which the subject chose C follow as;

$$CR_{i}=\frac{\#\left\{t\;\;:a_{i}\left(t\right)=Coop\right\}}{T},T=40$$

Individual defection rate was defined analogously as the proportion of trials with D (or equivalently 1−CR). These subject-level rates were used to characterize the overall distribution of cooperative/defective tendencies and to provide a baseline for subsequent dyadic and triadic metrics (Fig. 6A-B).

**Pairwise (mutual) and triadic cooperation rates** (Fig. 6A–B): To quantify cooperation at higher social scales, we computed dyad- and group-level indices [12]. Pairwise (mutual) cooperation rate. For each of the three dyads within a group (S1–S2, S1–S3, S2–S3), pairwise mutual cooperation rate was computed as the proportion of trials in which both members of the dyad simultaneously chose C as

$$MCR_{ij}=\frac{\#\left\{t\;\;:a_{i}\left(t\right)=Coop\wedge a_{i}\left(t\right)=Coop\right\}}{T},T=40$$

This yielded 33 dyad-level observations (3 dyads × 11 groups). When a subject-level pairwise index was required, dyad-level values were averaged across the two dyads involving that subject. Triadic cooperation rate. Triadic cooperation rate was defined as the proportion of trials with a CCC outcome (all three members chose C simultaneously), computed per group (N_g_=11).

**Trial-by-trial cooperation dynamics** (Fig. 6C): To visualize within-session dynamics, trial-wise cooperation was computed as the mean of 1[($a_{i}\left(t\right)=Coop$] across subjects at each trial [12]. The displayed trajectory used a 5-trial rolling mean to reduce noise and the SEM across subjects to represent uncertainty. The early/late split shown in Fig. 6C was used for descriptive visualization; corresponding summary statistics are reported in Table 1.

**Post-outcome transition probabilities: reciprocity and recovery** (Fig. 6D): We quantified history-dependent updating using conditional cooperation probabilities based on the previous trial’s joint outcome within a focal dyad [12]. For each subject, transition probabilities were computed from consecutive trial pairs (t−1 → t). Specifically, we computed:

Reciprocity: $P(C_{t}\;|CC_{t-1})$, the probability of choosing C at trial t given that both members of the focal dyad chose C at trial t−1.

Recovery: $P(C_{t}\;|DD_{t-1})$, the probability of choosing C at trial t given that both members of the focal dyad chose D at trial t−1.

Dyad-level transition probabilities were computed separately for each opponent and then aggregated to a subject-level estimate by averaging across the subject’s two dyads.

**Retaliation, forgiveness, and switching** (Fig. 6E–G): We computed additional strategy indices to capture asymmetric responses to the opponent(s)’ prior actions and action switching tendencies [12]. Retaliation (Fig. 6E): Retaliation was operationalized as increased defection following an opponent defection:

$P(D_{t}\;|oppD_{t-1})$compared against $P(D_{t}\;|oppC_{t-1})$ where $oppD_{t-1}$and $oppC_{t-1}$ indicate whether the opponent in the focal dyad defected or cooperated at trial t−1.

Forgiveness (Fig. 6F): Forgiveness was operationalized as cooperation following one’s own defection under an opponent-cooperative context: $P(C_{t}\;|ownD_{t-1},oppC_{t-1})$ compared against the baseline $P(C_{t}\;|ownD_{t-1})$.

Switch rates (Fig. 6G): Switching was quantified as action changes across consecutive trials: C→D switch rate and D→C switch rate, computed from the subject’s own consecutive actions.

As above, opponent-conditioned indices were computed per dyad and then averaged across the two dyads to obtain subject-level indices.

**Statistical tests and handling of missing conditioning events** (Table 1): All panel-wise comparisons in Fig. 6D–G were assessed using paired-samples t-tests at the subject level (two-tailed, α=0.05) [13]. For each subject, each conditional probability was computed using only transitions in which the required conditioning event occurred. If a subject never experienced the conditioning event (denominator = 0), the conditional probability was undefined and treated as missing; subjects were excluded pairwise for that comparison. Consequently, the effective sample size (Valid N) varies across panels. All test statistics (Valid N, df, t, p) and descriptive summaries are reported in Table 1.

### 4.8 Behavioral validation: computation of behavioral indices

**Fig. 7:**
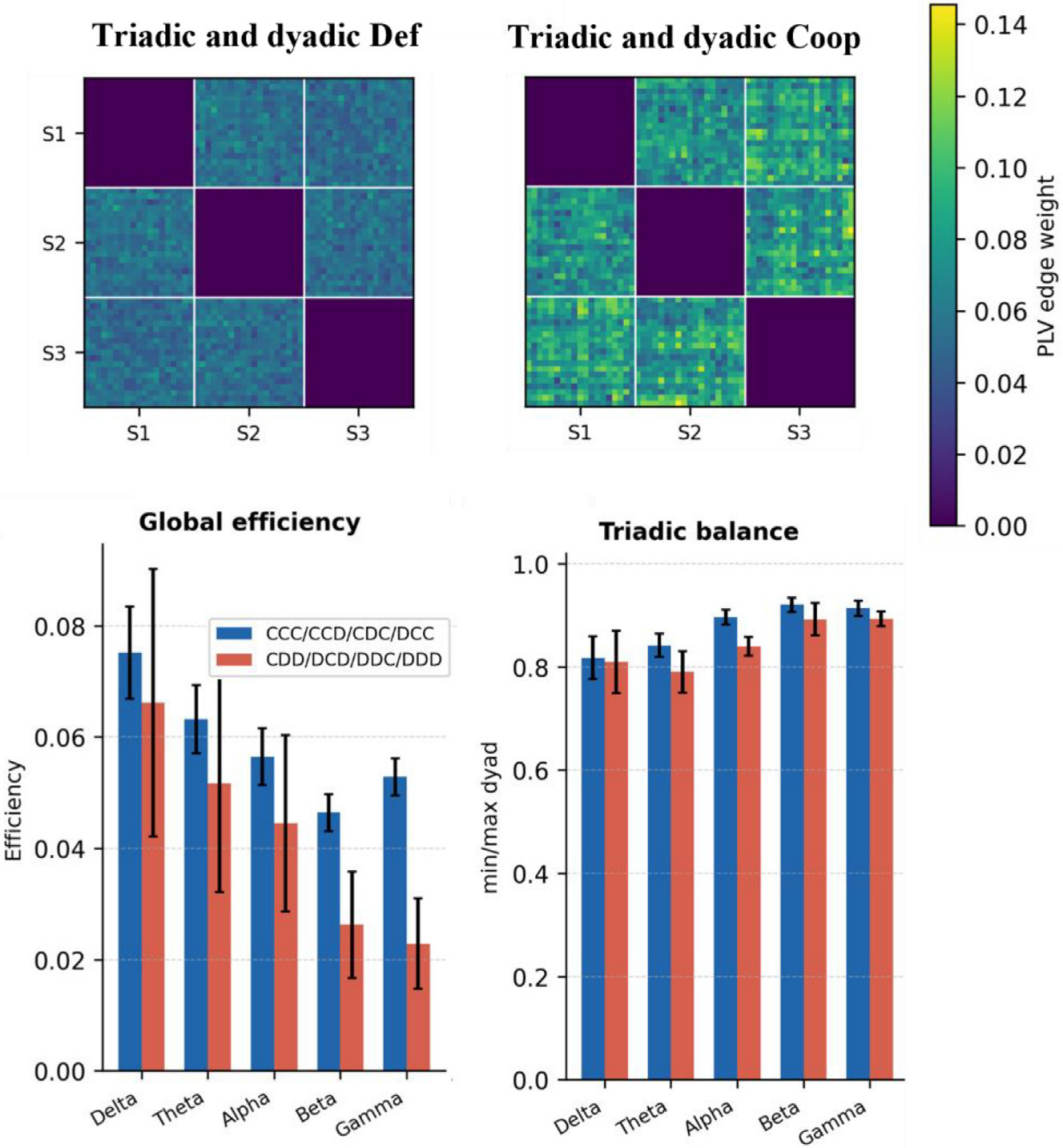
Triadic multilayer inter-brain network validation. (A–B) Condition-averaged supra-adjacency matrices representing PLV-based inter-brain connectivity among three participants. Each participant constitutes one layer containing 19 EEG electrodes, resulting in a 57 × 57 weighted matrix. White lines indicate the boundaries among the S1, S2, and S3 layers, and the diagonal within-participant blocks were set to zero. Matrices are shown for cooperation-dominant trials, comprising CCC, CCD, CDC, and DCC outcomes, and defection-dominant trials, comprising CDD, DCD, DDC, and DDD outcomes. (C) Weighted global efficiency calculated over the complete triadic supra-network. (D) Triadic balance, operationally defined as the ratio of the minimum to the maximum mean dyadic PLV among the three participant pairs.

To demonstrate the group-level analytical utility of the dataset beyond separate dyadic comparisons, we constructed a weighted triadic supra-network for each triad and experimental condition [14]. Each of the three participants was represented as one network layer containing 19 EEG electrodes, resulting in a 57-node multilayer representation. The off-diagonal blocks of the supra-adjacency matrix contained inter-brain PLV values for the three participant pairs, S1–S2, S1–S3, and S2–S3. The diagonal within-participant blocks were set to zero because the present validation focused on inter-brain rather than intra-brain connectivity (Fig. 7).

Trial outcomes were divided into cooperation-dominant trials, in which at least two participants selected cooperation (CCC, CCD, CDC, or DCC), and defection-dominant trials, in which at least two participants selected defection (CDD, DCD, DDC, or DDD). Condition-specific supra-adjacency matrices were generated separately for each triad and frequency band.

Two group-level network measures were calculated. Weighted global efficiency was computed over the complete 57-node supra-network to quantify the efficiency of information integration across the three participant layers. In addition, an operationally defined triadic balance index was calculated as the ratio between the minimum and maximum mean dyadic PLV values among the three participant pairs

The index ranges from 0 to 1, with values closer to 1 indicating that inter-brain coupling was distributed more evenly across the three dyads and lower values indicating that coupling was concentrated more strongly in one or two dyadic relationships. Although individual inter-layer edges were estimated from participant pairs, both global efficiency and triadic balance were calculated jointly from the three-participant interaction structure. Each triad was treated as one independent observational unit for group-level summaries and statistical comparisons (N = 11).

## Limitations

This dataset has several limitations related to sample size, data collection, and reuse. Although the dataset includes 33 participants, the independent inferential unit for group-level and triadic analyses is the triad, resulting in a group-level sample size of 11. Accordingly, statistical analyses of group-level correlations, network properties, and between-triad variability have limited power and may be sensitive to the behavior or signal characteristics of individual triads. The dataset is therefore most appropriate for methodological development, reproducibility assessment, exploratory analysis, and benchmarking of triadic hyperscanning methods, rather than for population-level normative inference. Future extensions should include a larger number of triads and participants from more diverse demographic and institutional backgrounds. Second, the experiment used a fixed triadic setting and a single iterated 3-player Prisoner’s Dilemma paradigm; therefore, the dataset does not cover other forms of group interaction, communication, or naturalistic social exchange. Third, EEG was recorded using dry-electrode devices, which can be more susceptible to contact variability and frontal artifacts, particularly around Fp1/Fp2. Channel-level quality information and preprocessing metadata are provided to support reuse. Finally, some behavioral transition events are sparse because cooperation was frequent in several groups, which may lead to missing conditional probabilities for specific transition-based behavioral metrics.

Synchronization relied on TCP/IP-based software event markers rather than a shared hardware clock or physical trigger. Although acquisition was monitored using a predefined 2-ms software-latency tolerance, the exact inter-device timing offset was not independently quantified using hardware-based validation.

## Ethics Statement

The study protocol was approved by the Institutional Review Board of Gwangju Institute of Science and Technology (GIST) (approval number: 20231207-HR-74-02-02). All participants provided written informed consent prior to participation. The study was conducted in accordance with the ethical principles of the Declaration of Helsinki. All released data were anonymized before public deposition, and no personally identifiable information is included in the shared dataset.

## CRediT Author Statement

**Heegyu Kim:** Conceptualization, Methodology, Software, Investigation, Data curation, Formal analysis, Writing – original draft. **Sung Chan Jun:** Investigation, Data curation, Writing – review & editing, Funding acquisition. **Chang S. Nam:** Conceptualization, Supervision, Writing – review & editing, Funding acquisition.

## Data Availability

FigshareEEG and Hyperscanning, triadic Prisoner's dilemma game (Original data)
